# Diagnostic value and safety of color doppler ultrasound-guided transthoracic core needle biopsy of thoracic disease

**DOI:** 10.1042/BSR20190104

**Published:** 2019-06-07

**Authors:** Weina Huang, Libin Chen, Ning Xu, Linfeng Wang, Fang Liu, Shiyi He, Tianwen Lai, Xueqin Chen

**Affiliations:** 1 Department of Respiratory and Critical Care Medicine, Ningbo First Hospital, 59 Liuting Road, Ningbo, China; 2 Department of Ultrasound, Ningbo First Hospital, 59 Liuting Road, Ningbo, China; 3 Department of Clinical Medicine, Medical School of Ningbo University, 818 Fenghua Road, Ningbo, China; 4 Department of Respiratory and Critical Care Medicine, The Affiliated Hospital of Guangdong Medical University, Zhanjiang, China; 5 Department of Traditional Medicine, Ningbo First Hospital, 59 Liuting Road, Ningbo, China

**Keywords:** Biopsy, Color Doppler ultrasound, Thoracic disease, Transthoracic Core Needle

## Abstract

**Objective:** The aim of the present study was to explore the diagnostic value and safety of color Doppler ultrasound (US)-guided transthoracic core needle biopsy (CNB) of peripheral lung, chest wall and mediastinal lesions using automated biopsy guns.

**Materials and methods:** We analyzed clinical and image data, histopathologic and microbiologic details and complications from 121 patients with peripheral lung, chest wall and mediastinal lesions who underwent color Doppler US-guided transthoracic CNB in Ningbo First Hospital between January 2015 and June 2018.

**Results:** Color Doppler US-guided transthoracic CNB performed with a freehand technique using automated biopsy guns had a sensitivity of 93.94%, a specificity of 100%, a positive predictive value of 100%, a negative predictive value of 78.57%, and a diagnostic accuracy of 95.04%. Lesion size did not affect the diagnostic rate (*P*=0.40). No serious complications of the procedure were noted.

**Conclusion:** Color Doppler US-guided transthoracic CNB of peripheral lung, chest wall and mediastinal lesions is a safe and inexpensive procedure. The diagnostic accuracy of color Doppler US-guided transthoracic CNB was higher than that of color Doppler US-guided transthoracic fine needle aspiration biopsy (FNAB).

## Introduction

As a result of advancements in technology, the imaging capabilities of color Doppler ultrasound (US) have been greatly improved. Currently, color Doppler US is a safe and efficient method for evaluating lesions of the peripheral lung, chest wall and mediastinum [1].

There may be insufficient material for examination with traditional bronchoscopic methods; it is difficult to obtain specimens with bronchoscopy from peripheral pulmonary and mediastinal lesions [2]. As reported in the literature, 40% of lung cancers present as peripheral masses that are potentially accessible to US [3]. Using color Doppler US to guide biopsy can provide real-time imaging of the procedure [4]. Core needle biopsy (CNB) is preferred over and superior to fine needle aspiration biopsy (FNAB) because CNB can obtain sufficient diagnostic specimens for histopathologic and cytological studies, and there is no significant difference in the complication rate between the two techniques [4–8].

Compared with computed tomography (CT)-guided transthoracic biopsy, color Doppler US-guided transthoracic biopsy has some advantages: a lack of exposure to radiation, a low complication rate, a relatively low cost and easily transported equipment [9].

The purpose of the present study was to explore the diagnostic value and safety of color Doppler US-guided transthoracic CNB of peripheral lung, chest wall and mediastinal lesions performed with a freehand technique using automated biopsy guns.

## Materials and methods

### Patients

The study included 121 patients who presented with peripheral lung, chest wall and/or mediastinal lesions by chest CT scan. Lesions were divided into nodules (≤3 cm) and masses (>3 cm) according to their maximum diameter in CT. The patients underwent color Doppler US-guided transthoracic CNB for histopathologic diagnosis in Ningbo First Hospital between January 2015 and June 2018. Some of patients also underwent color Doppler US-guided transthoracic CNB for microbiologic diagnosis. We extracted information on patient characteristics from electronic clinical records, including clinical, image data, histopathologic and microbiologic details and complications. The study protocol was reviewed and approved by the local ethics committee for Ningbo First Hospital in June 2018.

### US-guided transthoracic CNB

All patients had undergone chest CT scan before the procedure. CT images were used as a guide to determine the location of lung, chest wall and mediastinal lesions. Color Doppler US-guided transthoracic CNB was performed based on the following criteria: (i) as preconditions for puncture, there was no tendency for bleeding, the platelet count should have been higher than 100000/μl, and activated partial thromboplastin time should have been normal; (ii) patients with peripheral lung and mediastinal lesions had to have an accessible US window; (iii) no definite histological diagnosis should have been obtained before procedure; (iv) consent for the procedure should have been received from the patients.

The color Doppler US examination was performed with a C5-1 transducer (Philips, America) using a Philips system. Patients were placed in an upright, lateral decubitus, prone or supine position according to the position that provided greater access to the lesion and the best safety profile. The size, location, depth and internal structure of each lesion were evaluated from grayscale images. The vascularity of each lesion was estimated by color Doppler sonography to prevent potential complications of hemorrhage during the biopsy procedure. After cleaning the skin, a biopsy was performed under local anesthesia (lidocaine 2%). The US probe was also sterilized. A 16- or 18-gauge Acecut automate needle (TSK, Japan) was used for freehand biopsy. While conducting biopsy guided by real-time color Doppler US, needle shaft and tip visualization could be observed on two-dimensional US, and vessels could be seen on color Doppler imaging during the whole procedure. This real-time visualization of color Doppler is very helpful for avoiding overlying vessels. When the entire needle was moved, needle tip visualization could be intensified, allowing for better avoidance of vascular structures. Biopsy samples were removed, and histopathologic and microbiologic examinations were performed. All patients were observed clinically for 24 h.

### Statistical analysis

Data are presented as means ± standard error of the mean (SEM). Comparison between different groups was done using Student’s *t* test for continuous variables and c2 test for categorical variables. The sensitivity, specificity, positive predictive value and negative predictive value was reported to evaluate the ability of color Doppler US-guided transthoracic CNB in thoracic disease. The analyses were conducted using SPSS version 21.0 (SPSS, IBM, New York), and all tests were two-sided with a significance level of 0.05.

## Results

### Characteristics of participants

As shown in Table 1, a total of 121 patients (83 male and 38 female; mean age: 62.59 ± 15.69 years) were included in the study. There were 98 malignant cases (69 male and 29 female) and 23 benign cases (14 male and 9 female). Among these patients, lesions were located in the lung in 103 cases, in the anterior mediastinum in 10 cases and in the chest wall in 8 cases. In the malignant group, lesions were located in the lung in 82 cases, in the anterior mediastinum in 9 cases and in the chest wall in 7 cases. In the benign group, lesions were located in the lung in 21 cases, in the anterior mediastinum in 1 case and in the chest wall in 1 case. We also analyzed the characteristics of patients between the malignant and benign groups. A significantly lower age was observed in the benign group than in the malignant group (49.65 ± 16.02 *vs.* 65.63 ± 14.04; *P*<0.001).

**Table 1:** Characteristics of participants

Characteristics	Total	Malignant	Benign
Age, years	62.59 ± 15.69	65.63 ± 14.04	49.65 ± 16.02
Sex (male, female)	83/38	69/29	14/9
Location (*n*, %)			
Right upper lobe	23 (19.00)	19 (19.39)	4 (17.39)
Middle lobe	7 (5.79)	4 (4.08)	3 (13.04)
Right lower lobe	26 (21.49)	23 (23.47)	3 (13.04)
Left upper lobe	22 (18.18)	18 (18.37)	4 (17.39)
Left lower lobe	25 (20.66)	18 (18.37)	7 (30.34)
Anterior mediastinum	10 (8.26)	9 (9.18)	1 (4.35)
Chest wall	8 (6.61)	7 (7.14)	1 (4.35)
Size of lesion (*n*, %)			
Nodule (≤3 cm)	24 (19.83)	16 (16.33)	8 (53.33)
Mass (>3 cm)	97 (80.17)	82 (83.67)	15 (65.22)

In 97 (80.17%) cases, the lesion measured >3 cm (mass); in 24 (19.83%) cases, the lesion measured ≤3 cm (nodule).

### Diagnostic accuracy

The results of the biopsy were divided into malignant, benign and nondiagnostic. Table 2 shows that a definitive diagnosis was obtained in 115 out of 121 (95.04%) cases. A total of 93 cases were diagnosed as malignant. Among the 93 malignant cases, the diagnosis was adenocarcinoma in 34 cases, squamous carcinoma in 26 cases, adenosquamous carcinoma in 2 cases, small cell carcinoma in 2 cases, metastatic tumor in 10 cases, malignant thymoma in 4 cases, malignant pleural mesothelioma in 3 cases, lymphadenoma in 3 cases, and other indeterminate cancers in 9 cases. Twenty-two cases were diagnosed as benign. Among the 22 benign cases, the diagnosis was inflammation in 9 cases, abscess in 4 cases, pulmonary cryptococcosis in 3 cases, tuberculosis in 2 cases, proliferation of fibrous tissue in 2 cases, amyloidosis in 1 case, and teratoma in 1 case. Furthermore, microbiologic studies revealed 1 case of *Staphylococcus aureus*, 1 case of *Nocardi*a, 1 case of *Pseudomonas aeruginosa* and 3 cases of *Cryptococcus*. The above cases were confirmed by effective treatment.

**Table 2:** Study patients by diagnosis

Final diagnosis	Number	%
*Malignant*	93	76.86
Non-small-cell	66	54.55
Adenocarcinoma	34/66	
Squamous carcinoma	26/66	
Adenosquamous carcinoma	2/66	
Non-small-cell^1^	4/66	
Small cell	2	1.65
Metastasis	10	8.26
Malignant pleural mesothelioma	3	2.48
Lymphadenoma	3	2.48
Thymic carcinoma	4	3.31
Other cancers	5	4.13
*Benign*	22	18.18
Tuberculosis	2	1.65
Abscess	4	3.31
Amyloidosis	1	0.83
Inflammation	9	7.44
Proliferation of fibrous tissue	2	1.65
Pulmonary cryptococcosis	3	2.48
Teratoma	1	0.83
*Nondiagnostic*	4	3.31
*Insufficient sample*	2	1.65
Total	121	100

1 The specific non-small-cell carcinoma subtype could not be determined.

There were six nondiagnostic cases, as shown in Table 3. In two of the nondiagnostic cases, the size of the sample was insufficient, while in the remaining four cases, the diagnosis was inconclusive according to the histopathologic and microbiologic examinations and clinical data. Subsequently, conclusive diagnoses were obtained with CT-guided CNB or surgical biopsy. Histopathologic and microbiologic examination revealed the presence of squamous carcinoma in one case, large cell neuroendocrine carcinoma in one case, pulmonary cryptococcosis in one case, metastasis in one case, and adenocarcinoma in two cases.

**Table 3:** Analysis of the patient subgroup with nondiagnostic or insufficient samples

Patient	Diagnosis	Size measured by CT(mm)	Location	Final diagnostic method	Final diagnosis
#55	Inflammation	48	Right lower lobe	CT-guided CNB	Squamous carcinoma
#61	Proliferation of fibrous tissue	27	Left lower lobe	Surgery	Adenocarcinoma
#68	Insufficient sample	42	Right lower lobe	CT-guided CNB	Metastasis
#96	Inflammation	80	Left lower lobe	CT-guided CNB	Large cell neuroendocrine carcinoma
#97	Insufficient sample	65	Right upper lobe	CT-guided CNB	Adenocarcinoma
#120	Proliferation of fibrous tissue	24	Left lower lobe	CT-guided CNB	Pulmonary cryptococcosis

As shown in Table 4, color Doppler US-guided biopsy performed with a freehand technique using automated biopsy guns had a sensitivity of 93.94%, a specificity of 100%, a positive predictive value of 100%, a negative predictive value of 78.57% and a diagnostic accuracy of 95.04%. Lesion size did not affect the diagnostic rate (*P*=0.40).

**Table 4:** Diagnostic accuracy of US-guided CNB including or excluding insufficient samples

*Variable*	Including insufficient samples	Excluding insufficient samples
Number (*n*)	121	119
Nondiagnostic	6	4
Sensitivity	93.94%	95.88%
Specificity	100%	100%
Positive predictive	100%	100%
Negative predictive	78.57%	84.62%
Diagnostic accuracy	95.04%	96.94%

### Safety

No serious complications of the procedure were noted. Very light hemoptysis after puncture was observed in three cases, two cases recovered after hemostatic treatment, and one case resolved spontaneously. Among the cases with hemoptysis after puncture, one case had hemoptysis before puncture. Pneumothorax was noted in two cases; one presented with partial pneumothorax that did not need any treatment, while the other presented with interstitial lung disease and was treated with chest tube drainage. In our study, no air embolisms were detected. The incidence of complications was 4.13% (5/121). All complications recovered soon after treatment or spontaneously. No life-threatening or severe complications occurred.

## Discussion

Transthoracic needle biopsy (TNB) has more than 100 years of history [10–12]. In recent years, TNB has been widely used for both histopathologic and microbiologic diagnoses. It is usually performed under the guidance of US or CT [2,13–15].

US is a flexible approach that can guide many biopsy procedures in the chest [16].

To our knowledge, this is the first study of color Doppler US-guided transthoracic biopsy entirely using CNB with a high diagnosis accuracy. Many studies have shown that color Doppler US-guided transthoracic FNAB is useful and safe in the diagnosis of peripheral pulmonary lesions [2,17]. In this retrospective observational study in patients with peripheral pulmonary, chest wall and mediastinal lesions, we performed color Doppler US-guided transthoracic CNB with a freehand technique using automated biopsy guns. As a result, we found that the diagnostic accuracy of color Doppler US-guided transthoracic CNB was higher than that of color Doppler US-guided transthoracic FNAB (95.04 *vs.* 89.6%) since CNB can obtain more and larger specimens than FNAB. Sufficient specimens not only contribute to diagnosis but also help to determine tumor types and perform cytological and microbiological studies. In addition, the incidence of complications of color Doppler US-guided transthoracic CNB was low. There were no significant differences in the complication rates observed between CNB and FNAB [4–8]. Furthermore, when we analyzed the characteristics of patients between the malignant and benign groups, we found that the age of the benign group was much lower than that of the malignant group.

Many studies have shown that US is as effective as CT in guiding the biopsy of peripheral lung lesions, and the diagnosis yield based on deterministic histology was between 84 and 95% according to different studies [1,9,18,19]. In this study, color Doppler US-guided transthoracic CNB had a sensitivity of 93.94%, a specificity of 100%, a positive predictive value of 100%, a negative predictive value of 78.57% and a diagnostic accuracy of 95.04%. The US-guided biopsy proved to be an excellent modality for peripheral pulmonary and pleural lesions approachable with US guidance, although many radiologists favor CT-guided TNB [4,16]. Middleton et al. [16] studied a group of 54 patients with peripheral pulmonary or pleural lesions who underwent US-guided biopsies, and their success rate was 96% (52/54). A series of studies have indicated that the success rate of US-guided mediastinal biopsies is high [20–23]. Heilo [20] reported a success rate of 84% in a group of 58 patients with anterior mediastinal masses. Chest wall lesions are particularly superficial and typically suited for US-guided biopsies. In the present study, we obtained a diagnostic accuracy of 100% in six cases with chest wall lesions.

As reported in the literature, US-guided biopsy has many advantages over CT in cases of lesions in the pulmonary periphery or mediastinum that are potentially approachable with US guidance [17,24]. When radiography is contraindicated, US has clear advantages. In addition, the absence of radiation exposure, low complication rates and costs, and the ability to be performed at bedside are other advantages of US. Most importantly, color Doppler US can provide real-time visualization and enable the needle shaft or tip to be visualized during the whole biopsy procedure. This ability is very helpful when biopsying small nodules. Lesion size does not seem to affect the diagnostic rate in US-guided biopsy [25,26]. However, CT-guided biopsy is not performed in real-time, and the diagnostic rate of small lesions may be affected by breathing movements and by where the lesions are located with respect to the ribs. In the present study, as reported in the literature, we found that lesion size did not significantly affect the diagnostic accuracy in US-guided biopsy.

The color Doppler US-guided transthoracic CNB procedure is very safe because of a number of factors: (i) the needle tip can be visualized in real time on color Doppler images; (ii) large vascular structures can be visualized easily and avoided to minimize damage; (iii) US enables real-time monitoring of lesion motion with respiration, which is very critical when the lesion is small.

No serious complications of the procedure occurred in the present study. The overall complication rate was 4.13% (5/121), which was less than 5%. In contrast, CT-guided biopsy has a higher rate of complications [27,28]. Mild hemoptysis and minimal pneumothorax account for most of the complications of US-guided biopsy [1]. In this study, only one case of interstitial lung disease was treated with chest tube drainage, and one case of partial pneumothorax resolved spontaneously. Mild hemoptysis was observed in three cases. Among them, one case had hemoptysis before puncture. No blood transfusion was required for hemoptysis.

US also has its limitations. Because the US wave does not pass through air, which would be required for visualization of central lesions, pneumothorax or an aerated lung, a US window is not possible for US chest examinations.

There were some limitations to the present study. First, this is a retrospective study. Second, a freehand biopsy technique was used in the study. Third, the present study only involved 121 patients with thoracic disease from just one center, and the sample size for subgroup analysis was insufficient. Thus, multicentered, randomized studies with larger sample sizes are required.

In conclusion, color Doppler US-guided transthoracic CNB of peripheral lung, chest wall and mediastinal lesions has become an indispensable diagnostic tool for histopathologic and microbiologic studies. The diagnostic accuracy of color Doppler US-guided transthoracic CNB was higher than that of FNAB. The incidence of complications was low. There were no significant differences in the complication rates observed between CNB and FNAB. US-guided transthoracic CNB is relatively low cost and involves no radiation exposure. Color Doppler US can provide real-time visualization and enable the needle shaft or tip to be visualized during the whole biopsy procedure. It should be used and considered the ‘gold standard’ for procedure guidance for lesions approachable with US guidance.

## Availability of data and materials

The datasets used and analyzed during the current study are available from the corresponding author on reasonable request.

## Abbreviations

CNB: core needle biopsy
CT: computed tomography
FNAB: fine needle aspiration biopsy
TNB: transthoracic needle biopsy
US: ultrasound
